# Posterior vault encephaloceles: from antenatal management to post-surgical follow-up—a cooperative study

**DOI:** 10.1007/s00381-025-06764-x

**Published:** 2025-02-24

**Authors:** Claudia Pasquali, Ulrich-Wilhelm Thomale, Alexandru Szathamri, Pier Aurelien Beuriat, Valentina Pennacchietti, Mélodie Anne Karnoub, Matthieu Vinchon, Federico Di Rocco

**Affiliations:** 1 Hôpital Femme Mère Enfant, Université de Lyon, INSERM 1033, Lyon, France; 2 Pediatric Neurosurgery Charité – Universitätsmedizin, Berlin, Germany; 3 Pediatric Neurosurgery Hôpital Roger Salengro, Lille, France

**Keywords:** Atretic cephalocele, Occipital meningocele, Cerebral malformations, Prenatal diagnosis, Developmental outcome

## Abstract

**Purpose:**

Encephalocele is a herniation of intracranial structures associated with a skull anomaly. In Western countries, posterior encephaloceles are more common than anterior encephaloceles and may occur in the parietal (parietal encephalocele, PE) or occipital (occipital encephalocele, OE) region. Although those entities are relatively common in pediatric neurosurgery, large clinical series are scarce, and their clinical outcomes are poorly documented in the literature.

**Methods:**

We retrospectively analyzed the clinical and radiological findings, post-operative long-term outcomes of consecutive patients diagnosed with posterior encephaloceles from 2010 to 2021 in 3 centers: Hôpital Femme Mère Enfant (Lyon, France); Hôpital Roger Salengro (Lille, France); and Charité Universitätsmedizin (Berlin, Germany).

**Results:**

We collected 79 observations, 46 PEs and 33 OEs. Cerebral anomalies were more common in OEs than PEs (15/33, 45% vs 7/46, 15%, *p* = 0.001). Vascular anomalies were more common in PEs than OEs (41/46, 88% vs 5/33, 15%). All children underwent a surgical correction of the malformation. CSF disorders requiring surgical management were present in 4/33 OEs and 4/46 PEs. During the mean follow-up of 33 months (12–160 months), 3 OEs patients died, and various degrees of psychomotor impairment were found in both entities: 18/33 (54%) OEs and 8/46 (17%) PEs (*p* = 0.005); however, in most cases of PE, developmental delay was mild.

**Conclusions:**

The clinical evolution in OEs is significantly more unfavorable than in PEs. However, even in case of PEs, psychomotor impairments are not uncommon. The presence of herniated cerebral tissue, hydrocephalus, and syndromic context increase the risk of developmental delay.

## Introduction

Congenital herniation of the intracranial content through focal skull defects are rare malformations, of which overall incidence is estimated around 1–5 per 10,000 births, trough with geographic variations.

**Fig. 1:**
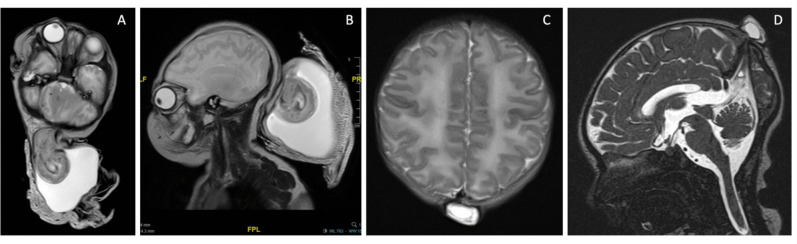
**A** T2-weighted MRI of an occipital encephalocele with herniation of cerebral and meningeal tissue, axial view (**A**); sagittal view (**B**); **C**–**D** T2-weighted MRI of a parietal meningocele characterized by the presence of meningeal tissue, liquid content and absence of cerebral tissue, axial view **C**), sagittal view (**D**)

In Western countries, their most frequent localization is the posterior skull with two main types: parietal and occipital ones. Occipital encephaloceles (OEs) occur between the lambda and foramen magnum, typically in the midline; parietal encephaloceles (PEs) originate from the lambdoid region with extension to the parietal region (Fig. 1) [1].

We can distinguish encephalocele, large lesion, epithelialized or not, containing identifiable central nervous system tissue; meningocele, skin-covered cystic lesions containing meninges and cerebrospinal fluid; and atretic encephalocele, a small epithelialized non-cystic lesion containing meninges and abortive CNS tissue, considered an abortive form of encephalocele [2].

Several theories have been postulated to elucidate their embryopathogenesis. The first theory considers encephaloceles as a neural tube defect (NTD) secondary to a failure of the closure of the anterior neuropore [3, 4]. However, recent studies speculate on the fact that, while anencephaly results from a failure of cranial neural tube closure, encephaloceles seem to derive primarily from a post-neurulation disease with a defect of cranial mesoderm development with a failure of separation between ectoderm and neuroectoderm [5–9]. This latter theory is supported by the observation in the same subject of the presence of both parietal and occipital encephaloceles as distinct entities [10], the coexistence of meningocele and dermal fistula in the same patient, and their occurrence in homozygous twins [11]. Some series have described genes implicated in the planar cell polarity signaling pathway like BMP, Sonic hedgehog, WNT, and in ciliopathies like MKS1-6 [12]. Mutations of ALK4 et MSX2, implicated in the regulation of skull vault ossification interacting with the FGFR, Twist1 and Runx2 pathways, have also been described in some cases [13, 14]. Encephaloceles can be isolated or be part of a genetic syndrome.

Unfortunately, the clinical outcomes of PEs and OEs are poorly and confusingly documented in the literature as well as the impact of possible associated anomalies accounting for the difficult prenatal counseling. The current study aims to provide useful findings in this direction.

## Materials and methods

We conducted a retrospective analysis of the clinical, radiological findings, and post-operative outcomes of patients with posterior cranial defects and herniation of intracranial content operated on at Hôpital Femme Mère Enfant (Lyon, France), Hôpital Roger Salengro (Lille, France), and Charité Universitätsmedizin (Berlin, Germany) between 2010 and 2021.

Defects located above the lambdoid region and within the parietal region were classified as parietal encephaloceles (PEs), while those between the lambdoid region and the foramen magnum were categorized as occipital encephaloceles (OEs). These malformations were further subdivided into encephaloceles, meningoceles, and atretic encephaloceles.

All patients underwent pre-operative MRI to assess the volume and type of herniated tissue, the size of the bone defect, and any associated parenchymal or vascular anomalies.

Descriptive statistical analyses were performed using Microsoft Excel (version 16.75), and univariate chi-square analysis was conducted with IBM SPSS Statistics (version 25.0). Statistical significance was set at *p* < 0.05.

The study was approved by the Institutional Review Board of Hospices Civils de Lyon under protocol number 00011687 on December 16, 2024.

## Results

A total of 79 patients were followed in the 3 centers for a posterior encephalocele (Table 1): 46 children presented with PEs and 33 with OEs. One subject with OE and 3 subjects with PE were preterm babies; all the remaining infants were born full term.

**Table 1:** Population and anatomical characteristics

	PE (46)	OE (33)	*p*
Number of patients (male:female)	46 26:20	33 13:20	0.13
Antenatal diagnosis	7 (15%)	20 (60%)	*p* < 0.001*
Age at surgical correction	7.5 months (9% first month of life)	6.5 months (32% first month of life)	
Different forms:			
- Atretic	36 (78%)	5 (15%)	
- Meningocele	8 (18%)	15 (45%)	
- Encephalocele	2 (4%)	13 (40%)	
Parenchymal and cranio-cerebral anomalies	7 (15%)	15 (45%)	*p* = 0.001*
Vascular anomalies	41 (88%)	5 (15%)	*p* = 0.001*
Hydrocephalus	2 (4%)	4 (12%)	*p* = 0.03*
Extracranial anomalies	6 (13%)	10 (30%)	ns

**Fig. 2:**
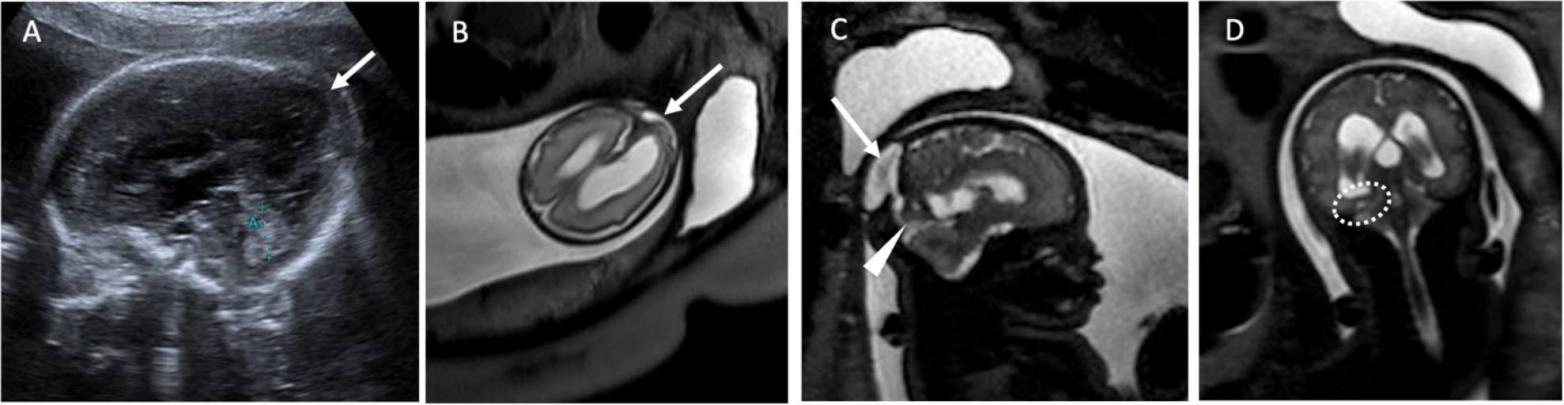
Twenty-six-week echography (**A**) and MRI (**B** axial view; **C** sagittal view; **D** coronal view) with the evidence of an encephalocele showing a parieto-occipital bone defect with a herniation of the cerebral hemisphere (white arrow). No anomaly of the posterior cerebral fossa was detected; however, there was a notable dehiscence of a large part of the cerebellar tent with (round in image **D**), potentially, a right sinus replaced by a falcoriel sinus corresponding to an accessory sinus rising towards the bone defect (point of arrow in image **C**). No major anomalies of cerebral organization except for small occipital subependymal heterotopias were described

The antenatal diagnosis (Fig. 2) was made in 27 patients: 20 patients with OEs (60% of the subgroup) and 7 with PEs (15% of the subgroup). This difference was statistically significant (*p* < 0.001) and probably reflected the fact that occipital encephaloceles tend to be larger, with a liquid component.

Most of the parietal malformations (78%) were atretic encephaloceles, 18% meningoceles, and 4% encephaloceles. In contrast, among the occipital malformations, only 15% were atretic encephaloceles while meningoceles represented 45% and properly defined encephaloceles 40% of the group.

The diameter of the malformation went from 1 to 2 cm for the parietal meningocele and from 1 to 3 cm for occipital meningocele; the diameter of occipital encephalocele went from 1 to 5 cm.

**Fig. 3:**
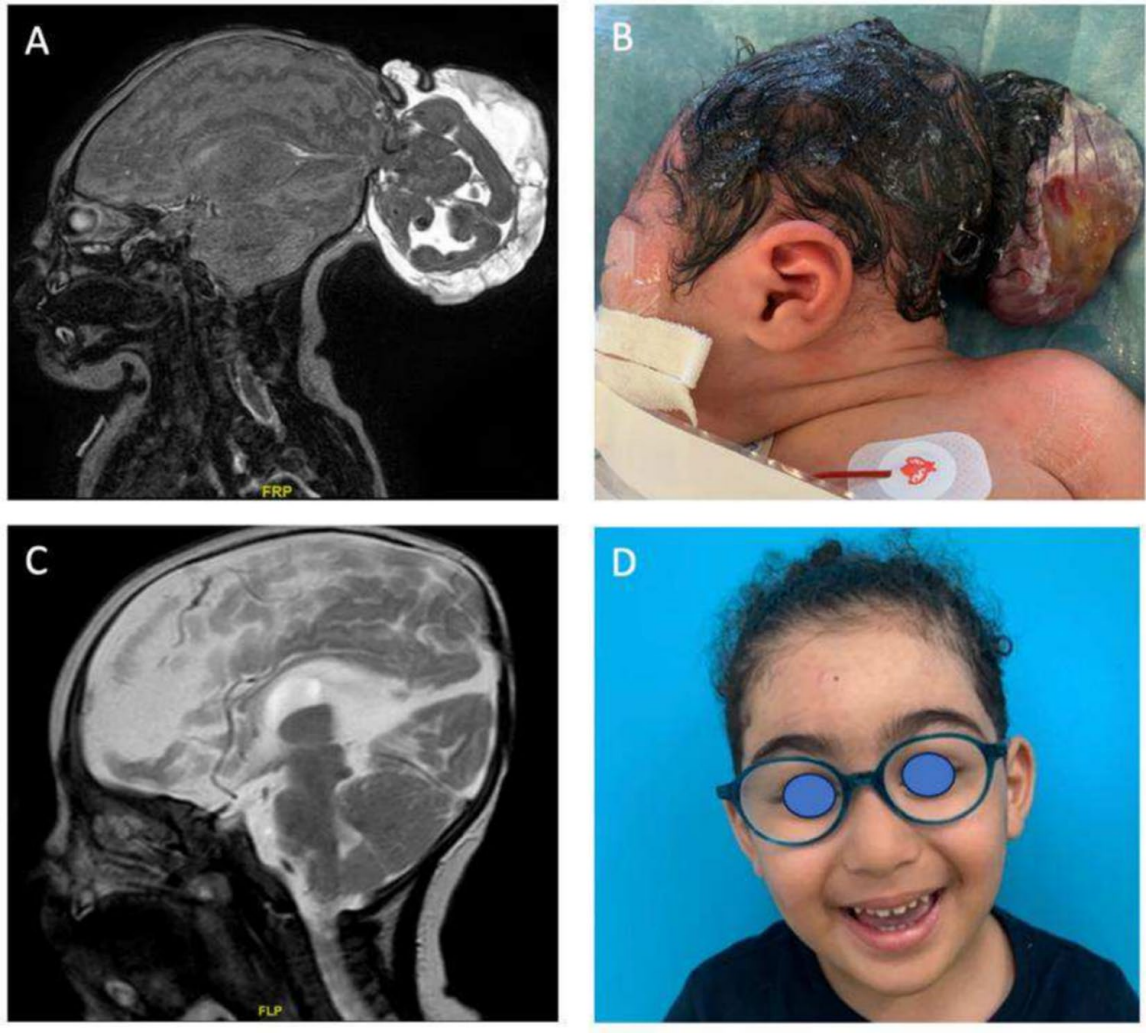
Occipital encephalocele, before and after surgical correction. **A** Postnatal sagittal T2-weighted MRI: we can appreciate the herniation of formed neural structure at occipital level; **B** pre-operative image of occipital encephalocele; **C** 5 years post-operative T2-weighted sagittal MRI showing the complete correction of the encephalocele; and **D** patient aspect at the follow-up

All the infants were treated surgically. The mean age at surgical correction was similar in the two groups: 6.5 months for the OEs and 7.5 months for the PEs. While only 9% (4/46) of PEs were operated in the first month of life, 11/33 (32%) infants with OEs required surgical correction within the first 4 weeks of age on account of large size of the herniation and in one case because of a non-epithelialized lesion (Fig. 3).

**Fig. 4:**
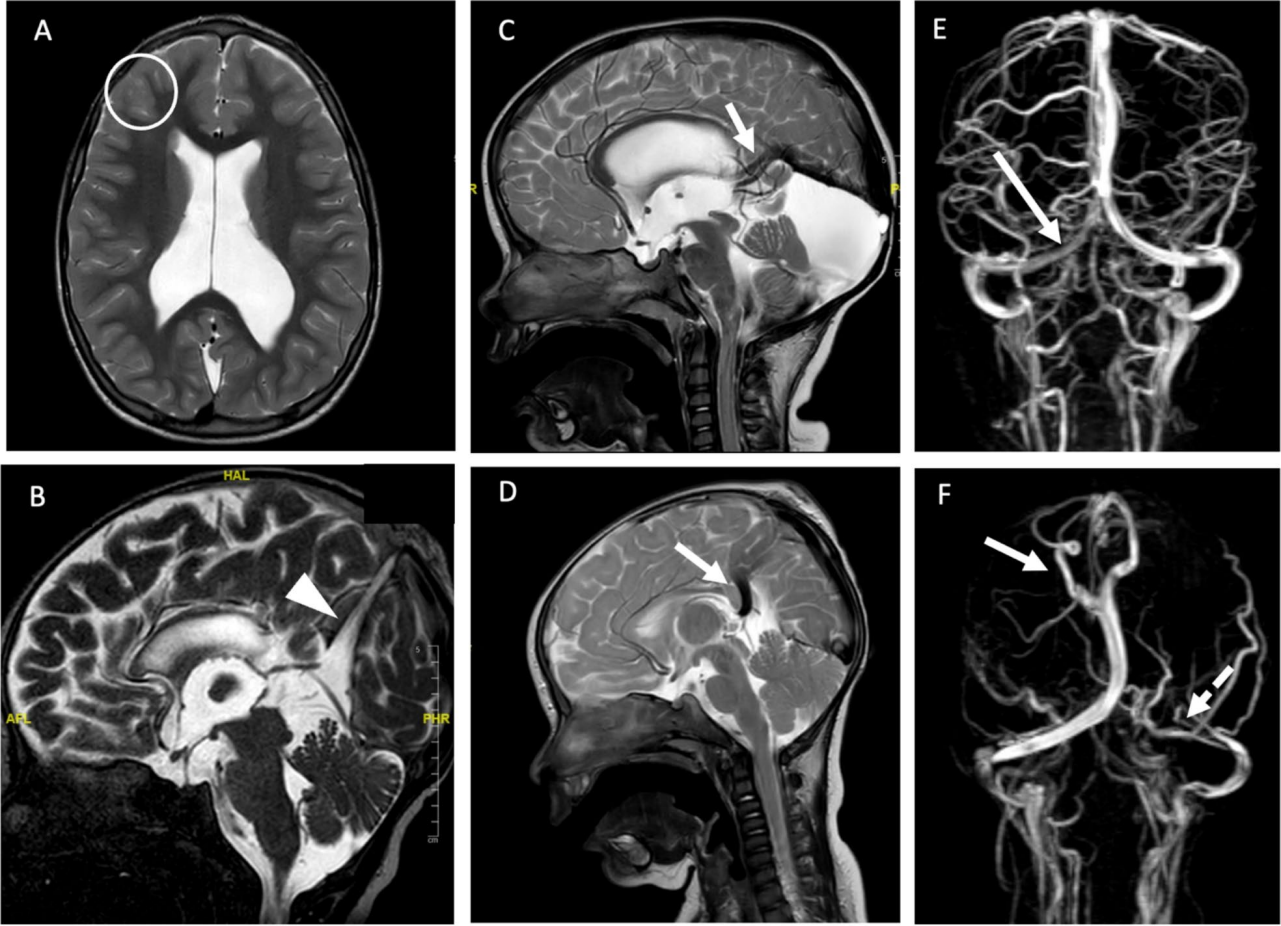
Some examples of cerebral and vascular anomalies. **A** Right frontal heterotopies in an OE patient (white round); **B** Hight tentorium implant in a PE patient (point of arrow); **C** sinus rectus duplication in a PE; **B** agenesis of sinus transverse in a PE (white arrow); **C** persistence of the vein falcine in a PE (white arrow); **D** aberrant drainage veins (white arrow) associated to the agenesis of left sinus transverse (dotted arrow) in an OE

Parenchymal anomalies and cranio-cerebral anomalies were found in 15/33 (45%) OEs and 7/46 (15%) PEs (*p* = 0.001) (Fig. 4A, B). These anomalies appear to be more severe in case of OEs, including different degrees of cranio-vertebral junction anomalies, rhombencephalosynapsis, periventricular heterotopies, gyration disorder, hypoplasia of corpus callosum, or vermis. Among the PEs group, the cerebral anomalies were mostly represented by a dysplastic corpus callosum, periventricular heterotopies, and Dandy-Walker malformation. In 2 cases, it was observed a synostosis of calvaria sutures (lambdoid in one case and sagittal in the other). Interestingly, more than 90% of patients (41/46) presented with some anomalies of cerebellar tentorium; mostly (75%, 35/46) an elevation of tentorium and in the other cases by its dehiscence and cerebellar hypoplasia was noticed in 15/46 (32%) of patients.

Vascular anomalies (Fig. 4C–F) were found in 12% of OEs and in 88% of PEs (*p* = 0.001). In OEs, these anomalies were particularly represented by anomalies of drainage veins, followed by agenesis of the transverse sinus, thrombosis of the transverse sinus, and duplication of straight sinus, while in the PEs persistent falcine vein was a common finding, along with the duplication of sinus rectus or the agenesis of transverse sinus, which generally do not have functional consequences or surgical implications.

Hydrocephalus requiring a surgical treatment occurred in 2 patients (4%) diagnosed with PE: one of them aged 1 month had a concomitant Dandy-Walker malformation and was managed some weeks after the correction of meningocele with a ventriculoperitoneal shunt, the second one had stenosis of the aqueduct and was treated endoscopically 5 months after the correction of the atretic encephalocele. Among those with OEs, 4 patients (12%) presented with hydrocephalus and received a ventriculoperitoneal shunt insertion between 3 and 6 months after the repair of encephalocele.

**Fig. 5:**
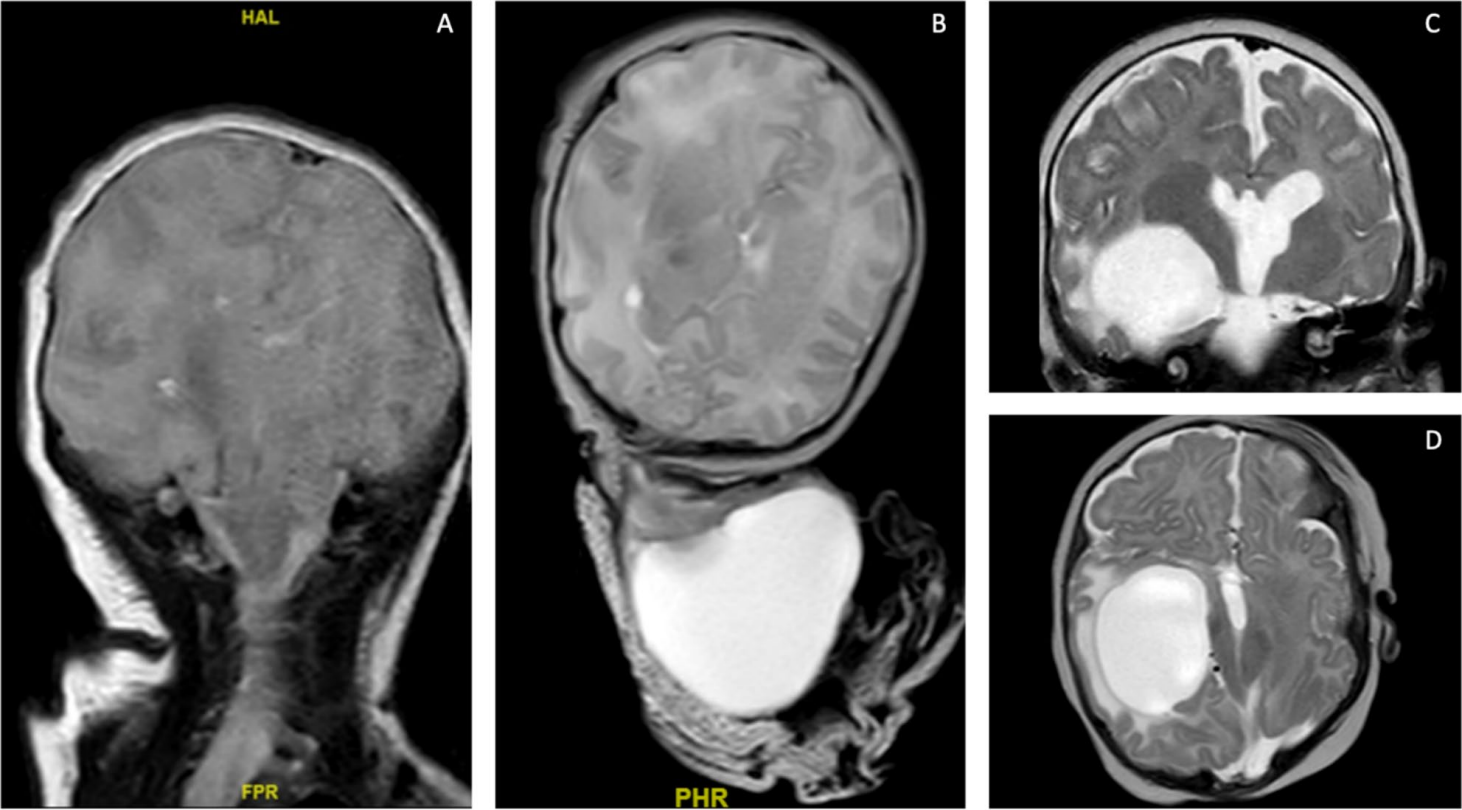
Postnatal, pre-surgical T2-weighted coronal (**A**) and T2-weighted axial (**B**) MRI: right temporal arachnoid cyst nonvisible. Post-operative control MRI 9 months after the surgical correction of the encephalocele, T2-weighted coronal (**C**), and T2-weighted axial MRI (**D**): development of ventriculomegaly and a right temporal arachnoid cyst with mass effect and deviation of the midline structures

Ventriculomegaly that did not require treatment was noted at the diagnosis in 2 patients with OEs and 1 patient with PE. Arachnoid cysts (one temporal and one cerebellar in the midline posterior cranial fossa) were diagnosed in 2 cases of OEs requiring an endoscopic cystostomy because of increasing volume at 9 months and 6 years after the surgical correction of encephalocele (Fig. 5).

Extracranial-associated anomalies of different degrees of severity were found in 30% of OEs and in 13% of PEs infants: cutaneous lumbar midline abnormalities (like angiomas and sacral dimples) without spinal dysraphism were found in 2/33 OEs (6% of subgroup) and in 1/46 PEs (2% of subgroup), long bone anomalies in 1/46 PEs (2% of subgroup), diffuse osseous anomalies associated to scoliosis in 1/33 OEs (3% of subgroup), facial dysmorphism was present in 2/46 PEs (4% of subgroup), and in 1/33 OEs (3% of subgroup).

Furthermore, one patient with PE had a VACTERL syndrome. Among OEs, there were patients with tracheal mass (1/33), severe lung dysplasia (1/33), renal anomaly (1/33), huge nasal angioma (1/33), and agenesis of internal ear (1/33).

### Surgical outcomes

Surgical complications occurred in 12% (4/33) of OEs: post-operative-nonsurgical hemorrhages in 2 cases; intraoperative bleeding in 1 case, forcing to abort surgery and requiring a redo surgery; cerebral edema in 1 case. Only 1 patient treated for a PE (2%) presented a post-operative hemorrhage that required a surgical intervention.

A healing problem of the surgical wound occurred in 2 patients with OE (6%), one of them requiring a second surgery for surgical revision. No PE patients presented with healing problems.

In the whole series, we did not record any post-surgical infection.

The diameter of persistent post-operative osseous lacunae went from sub-centimetric to 4 cm. Bone defects were larger (with a mean diameter of 2 cm) in patients with OEs than PEs patients (with a mean diameter of 0.7 cm).

A cranioplasty was necessary in 2 patients treated for PEs (4%) respectively: 3 years later, the surgery for the correction of the encephalocele in one patient and 5 years later the correction of an atretic encephalocele in the other case. In the OEs group, 2 (6%) patients treated at birth for encephaloceles needed a cranioplasty: 1 year later in the first case because of a huge defect stable at control and 4 years later in the other case because of the progression of the bone defects with the characteristics of a growing fracture.

Depending on the surgeon’s habits, both autologous cranioplasties using split calvaria and cranioplasties using heterologous material were performed in 2 cases each.

### Clinical outcomes (Table 2)

**Table 2:** Clinical outcomes

	PE (46)	OE (33)	*p*
Psychomotor impairment	8 (17%)	18 (54%)	*p* = 0.005
Psychomotor impairment at neuropsychological test			
- Atretic	2/18 (11%)	0/3 (0)	
- Meningocele	1/4 (25%)	4/6 (67%)	
- Encephalocele	1/1 (100%)	8/10 (80%)	
Psychomotor impairment			
- With hydrocephalus	2/2 (100%)	4/4 (100%)	ns
- Without hydrocephalus	6/44 (13%)	14/29 (48%)	*p* = 0.003*
School adaptation	12 (25%)	12 (37%)	
Visual disorders	0	11 (32%)	
Death	0	2 (9%)	

At a mean follow-up of 33 months (range 12–160 months), delayed milestones and psychomotor impairment were noticed in 54% (18/33) of OEs and 17% (8/46) of PEs subjects (*p* = 0.005) requiring school adaptation for 37% of OEs and for 25% of PEs children.

In particularly, in a subgroup older than 1 year, evaluated by neuropsychological tests composed of 23 PE and 19 OE, psychomotor disorders were observed in one case (1/1) of encephalocele, in 1 out 4 of meningocele and 2 out 18 subjects with atretic forms in parietal location. In the occipital location, psychomotor disorders were present in 8 out 10 children with encephalocele, in 4 out 6 cases of meningocele, and in no subject with the atretic form (0/3). Furthermore, in the case of atretic form with psychomotor disturbances in parietal region, one patient was syndromic, and the second one was suffering from severe hydrocephalus that required surgical treatment.

Focusing on the 6 patients (4 OEs, 2 PEs) treated for hydrocephalus, all manifested delayed psychomotor development regardless of the location and the volume of the encephalocele.

During the follow-up 3 patients, all with OE, died: 2 for intercurrent pathologies and 1 because of a severe encephalopathy in a syndromic context.

## Discussion

The improved medical and surgical management of infants with cerebral malformation resulting in current higher survival rates is confirmed by the analysis of our series of infants with posterior encephaloceles where we recorded no perioperative mortality nor infection. Surgical complications were relatively few in our series: 4 OEs and 1 PE patients had ischemic-hemorrhagic complications requiring surgical reintervention in 2 cases, 1 required revision of surgical wound, and no mortality directly related to surgery was registered.

Three late deaths for intercurrent diseases or complications related to the syndromic context, were observed in the OEs group resulting in a global death rate of 15.1% in this subgroup.

The mortality rate is variable in the surgical series: from 0 [15] to 33% in PE [16], and in the case of OE from 2% [17] up to 30% [18–20] with peak to 60% within the first year of life in series including giant lesions [21–23].

The main causes of mortality and morbidity were the perioperative hemorrhage and post-operative infection.

In our study, 2 OE patients required a redo surgery because surgery was aborted on account of hemorrhagic complication during the first surgery; one of them presented with severe post-operative cerebral edema. The absence of such complications in PE can be explained by the larger prevalence of impaired venous drainage in OEs than PEs, the latter presenting mostly with anatomical variants without clinical impact, like the persistence of falcine vein or the duplication of sinus rectus.

In recent years, the reduction of intraoperative and post-operative complications has been possible thanks to high resolution imaging which permits an adequate surgical planning allowing to identify associated vascular anomalies, the type of herniated tissue, and the improvement of perioperative care [22].

In most cases of PEs and in infants with atretic encephaloceles, the operation has mainly a cosmetic purpose. In patients with OEs, besides the curative purpose, early surgical treatment is considered to have a preventive role by avoiding complications such as rupture, ulceration, or infection of the lesion and, more importantly, the increased damage of the brain due to its progressive herniation [2]. In case of progressive herniation, the skull becomes hypoplastic leading to microcrania unable to accommodate further brain growth in case of tardive surgical correction. In fact, a recent study has claimed that operations performed in utero are associated with a significant better neurologic outcomes than those possible with postnatal repair of the malformation by precociously counteracting the microcephaly secondary to cerebral displacement outside of the skull [24]. The relevance of such an experience, when confirmed by further observation, is particularly obvious at the light of the recent technical progresses for the prenatal diagnosis since the recognition of the OEs is nowadays possible as soon as week 12th of gestation [19]. In OEs cases, the antenatal recognition of the lesion is favored by its increased size and liquid content so explaining that the prenatal diagnosis of the OEs represents up to 70% of the antenatal diagnosis of posterior encephalocele [25].

Despite early surgical repair performed during the first months of life in all the cases of our series, the long-term outcomes were not significantly different from those described in previous reports, by excepting the higher mortality rate.

Psychomotor impairment at long-term follow-up was noticed in 18/33 (54%) OEs versus 8/46 (17%) PEs. These results are at odds with those reported by Yokota et al., on 29 cases (14 OEs whose 5 atretic forms, and 15 PEs, whose 5 atretic form) who also stressed the worse clinical outcome of OEs compared to PEs. In the same study, only 16% of infants with OEs achieved normal development versus 54% of the subjects with PEs. [16]

In literature, it has been found a correlation between the degree of brain tissue herniation and severity of the subsequent impairment psychomotor development. More vascular anomalies do not seem to predict a poor outcome [26, 27] in contrast with hydrocephalus, seizure disorder, microcephaly, associated cerebral abnormalities, and brain tissue herniation which are significantly correlated with poor outcome in univariate analysis. Hydrocephalus and intracranial abnormalities are independent prognostic factors in multivariate analysis [28, 29].

The development of post-operative hydrocephalus should be regarded as the major complication of operated OEs which requires surgical management up to 60% of the cases [30] and carries on a high risk of infection and malfunction of the inserted CSF shunt device observed in 8 to 20% of the cases with a peak of dysfunction up to 90% in the first 2 years following the surgery [31, 32].

Associated cerebellar anomalies, such as, for example, Dandy-Walker malformation, can contribute to the intrinsic risk of developing hydrocephalus in subject with posterior encephaloceles. A literature review of 20 years carried out by Talamonti in 2011 allowed to collect 30 cases of such association with hydrocephalus requiring CSF diversion in 90% of patients [33]. We observed only one case of parietal meningocele (treated at 1 month of age) with associated Dandy-Walker malformation who needed ventriculoperitoneal shunts after the correction of meningocele because of evolutive hydrocephalus.

Other risk factors for post-surgical hydrocephalus such as microcephaly, preexisting ventriculomegaly, occipital localization, and non-atretic forms of meningocele are described in the literature [32, 34]. In our series, no preexisting ventriculomegaly (1 atretic PEs; 2OEs: 1 encephalocele, 1 meningocele) required any treatment.

In our series, a small percentage of patients required shunt after the surgical correction (4/33 OE and 2/46 PE) and no patient needed shunt revision during the follow-up period.

None of our patients with an atretic encephalocele underwent treatment for hydrocephalus, in accordance with the literature.

Cranioplasty was required in 4 of our patients (2/46 PEs and 2/33 OEs); it is reportedly more frequently needed in OE than in PE, reflecting the presence of large herniation of cerebral content through an osseous defect in that group. These data are in line with literature reporting an incidence of cranioplasty up to 9% in mixed series, also including anterior encephaloceles, reflecting the fact that generally the cosmetic results are good [34].

Concerning long-term outcome, our experience confirms the data in the literature; the occipital localization bears a significantly worse prognosis than the parietal one with psychomotor impairment occurring in 18/33 (54%) OEs versus 8/46 (17%) PEs.

Since hydrocephalus is clearly associated with a worse prognosis, it is worth noting that even when excluding such a complication, in our patients, OEs still had a worse long-term outcome (14/29 (48%)) than PEs (6/44(13%)) with a statistically significant difference (*p* = 0.003).

With regards to properly defined encephaloceles, 9 cases in our series, 1/1 in the PEs group and 8/10 in the OEs group, presented some degree of psychomotor impairments, further stressing the negative impact of neural tissue herniation.

## Conclusion and implications for the prenatal management

PEs and OEs are distinct entities, regarding their associated lesion, management, and prognosis. Both the diagnosis of congenital encephalocele and the distinction between PEs and OEs during the antenatal period are fundamental to provide accurate information and adapted management for patients and parents.

The clinical evolution is significantly more favorable in the case of PEs compared to OEs where psychomotor disorders at follow-up are diagnosed in 54% of patients. However, although PEs have a reputation of having a benign clinical outcome particularly in their atretic form, they still are at risk of psychomotor impairment, which was present in 17% of our patients at follow-up.

The prevalence of psychomotor impairment rises steeply when intracranial abnormalities (in 45% of OEs and 15% of PEs) or hydrocephalus (12% of OEs and 4% of PEs) are present. Considering the paramount prognostic importance of associated anomalies (herniated cerebral tissue, hydrocephalus, and syndromic context), the most detailed prenatal imaging is warranted, regarding of the central nervous system, but also other organs.

Atretic encephalocele, which is rarely diagnosed prenatally, does not require specific obstetrical management, while large meningoceles and encephaloceles may require cesarean section and birth in an institution with pediatric neurosurgical facilities.

## Data Availability

No datasets were generated or analysed during the current study.
