# Correction: Posterior vault encephaloceles: from antenatal management to post-surgical follow-up—a cooperative study

**DOI:** 10.1007/s00381-025-06873-7

**Published:** 2025-06-14

**Authors:** Claudia Pasquali, Ulrich-Wilhelm Thomale, Alexandru Szathamri, Pier Aurelien Beuriat, Valentina Pennacchietti, Mélodie Anne Karnoub, Matthieu Vinchon, Federico Di Rocco

**Affiliations:** 1 Hôpital Femme Mère Enfant-Centre de Référence Craniosténose, Université de Lyon, INSERM 1033, Lyon, France; 2 Pediatric Neurosurgery Charité – Universitätsmedizin, Berlin, Germany; 3 Pediatric Neurosurgery Hôpital Roger Salengro, Lille, France

**Correction: Child's Nervous System (2025) 41:121**

10.1007/s00381-025-06764-x

In this article the author’s name 'Ulrich-Wilhelm Thomale' was incorrectly written as 'Urlich Thomale'. Also, an incorrect article type was selected; the article is not a 'Review', but an 'Original Research' article.

The original article has been corrected.

